# Directed Networks as a Novel Way to Describe and Analyze Cardiac Excitation: Directed Graph Mapping

**DOI:** 10.3389/fphys.2019.01138

**Published:** 2019-09-10

**Authors:** Nele Vandersickel, Enid Van Nieuwenhuyse, Nico Van Cleemput, Jan Goedgebeur, Milad El Haddad, Jan De Neve, Anthony Demolder, Teresa Strisciuglio, Mattias Duytschaever, Alexander V. Panfilov

**Affiliations:** ^1^Department of Physics and Astronomy, Ghent University, Ghent, Belgium; ^2^Department of Applied Mathematics, Computer Science and Statistics, Ghent University, Ghent, Belgium; ^3^Computer Science Department, University of Mons, Mons, Belgium; ^4^Ghent University Hospital Heart Center, Ghent University, Ghent, Belgium; ^5^Department of Data Analysis, Ghent University, Ghent, Belgium; ^6^Cardiology Department, AZ Sint-Jan, Bruges, Belgium; ^7^Laboratory of Computational Biology and Medicine, Ural Federal University, Ekaterinburg, Russia

**Keywords:** network theory, cardiac arrhythmia, atrial tachycardia, rotational activity, focal activity, phase mapping

## Abstract

Networks provide a powerful methodology with applications in a variety of biological, technological and social systems such as analysis of brain data, social networks, internet search engine algorithms, etc. To date, directed networks have not yet been applied to characterize the excitation of the human heart. In clinical practice, cardiac excitation is recorded by multiple discrete electrodes. During (normal) sinus rhythm or during cardiac arrhythmias, successive excitation connects neighboring electrodes, resulting in their own unique directed network. This in theory makes it a perfect fit for directed network analysis. In this study, we applied directed networks to the heart in order to describe and characterize cardiac arrhythmias. Proof-of-principle was established using *in-silico* and clinical data. We demonstrated that tools used in network theory analysis allow determination of the mechanism and location of certain cardiac arrhythmias. We show that the robustness of this approach can potentially exceed the existing state-of-the art methodology used in clinics. Furthermore, implementation of these techniques in daily practice can improve the accuracy and speed of cardiac arrhythmia analysis. It may also provide novel insights in arrhythmias that are still incompletely understood.

## 1. Introduction

One of the most effective ways to treat atrial and ventricular tachycardias is catheter ablation. In most of the cases ablation is guided by activation maps obtained from electroanatomical mapping systems. From these maps electrophysiologists need to precisely determine the mechanism of an arrhythmia—focal or reentrant—and assess the conduction pattern for a given patient to choose the proper ablation strategy. Performing it in complex substrates often confronts electrophysiologists with uncertainty (Delacretaz et al., 2001; Rostock et al., 2010; Kaiser et al., 2018; Martin et al., 2018). In these cases, the ablation procedure tends to be complex and time-consuming. This is particularly true for atrial tachycardias (AT) occurring after surgery or prior ablations (i.e., after ablation of persistent atrial fibrillation, Delacretaz et al., 2001; Deisenhofer et al., 2006; JaÏs et al., 2006; Patel et al., 2008; Rostock et al., 2010) and for scar-related ventricular tachycardias (VT) (Martin et al., 2018). If an incorrect target is ablated, not only will the patient not be cured, but new arrhythmias may be induced due to scarring (Chugh et al., 2005; Deisenhofer et al., 2006). In order to optimize catheter ablation, new tools for assessment of cardiac excitation patterns are needed to determine the underlying mechanism and to help identify the correct ablation target.

In the present study we propose a novel approach based on directed networks which allow the automatic determination of the type of cardiac arrhythmia (rotational or focal) and the characterization of important features of the excitation pattern which can be used for the automatic guiding of the ablation strategy. A network, in the most general sense, is a collection of nodes connected by links, which can represent diverse systems. Over the past 20 years, network theory has had many applications, ranging from biology to social sciences (Barabási, 2016). Examples include the PageRank algorithm (Brin and Page, 2012) for the World Wide Web which formed the basis of Google; determining the shortest route(s) between two places; modeling of molecules (e.g., fullerenes, Kroto et al., 1985), social networks (Borgatti et al., 2009), interactions of genes, proteins, metabolites and other cellular components (Barabasi and Oltvai, 2004; Barabási et al., 2011); the spread of diseases (Danon et al., 2011; Brockmann and Helbing, 2013); and many others. More recently, networks led to the development of novel diagnostic biomarkers in Alzheimer's disease, multiple sclerosis, traumatic brain injury and epilepsy (Stam, 2014). A network can be directed or undirected depending on if the connecting links have a direction from one node to the other. In spite of this variety, directed networks have not yet been applied to identify the sources of cardiac arrhythmias in the heart.

Directed networks naturally occur in the analysis of excitation patterns recorded by electrodes. When connecting discrete points of measurement in proximity to each other based on their local activation times (LAT), a directed network is created. This network of cardiac excitation appears suitable for directed network analysis. By applying network theory, conduction paths can be identified in a new and different way based on local activation times and by taking the physiological conduction velocity into account. During the analysis, the algorithm identifies potential ablation targets such as rotational activity, spreading from electrode to electrode creating a closed loop, or focal activity, manifesting as a divergence of excitation from a given point (region). We refer to this method as directed graph mapping (DG mapping). In graph theory, very efficient methods have been developed to find closed loops in directed networks. Using these methods, one can easily find all possible loops in these data within mere seconds. Since this approach analyzes all possible loops automatically, it forms a robust method even in the presence of noise or incorrect electrode recordings, making it much more reliable than current existing methods. Furthermore, it allows the determination of additional properties of excitation as well, which can be essential for the characterization of the arrhythmia. By using directed networks and DG mapping, we believe that a more reliable, faster and fully automatic analysis of activation patterns can be performed with a higher accuracy than in current daily practice.

The goal of this study is to demonstrate the wide applicability of directed networks to the heart for each driving mechanism of cardiac arrhythmias in both the atria and the ventricles. Therefore, we tested the accuracy of DG mapping in *in-silico* (ventricular) models of functional and anatomical reentry and focal activity. To determine the accuracy of DG mapping in the atria, we analyzed 31 clinical cases of atrial tachycardia. Regular AT is a clinical tachyarrhythmia in which the operator can be sure about the location of the tachycardia since ablation of the correct target almost always results in immediate success. Therefore, AT was used as the gold standard for validating DG mapping in a clinical setting. In addition, DG mapping was compared to phase mapping (Gray et al., 1998) via *in-silico* simulations, a widely used technique for detecting the center of a rotor.

## 2. Materials and Methods

**Figure 1:**
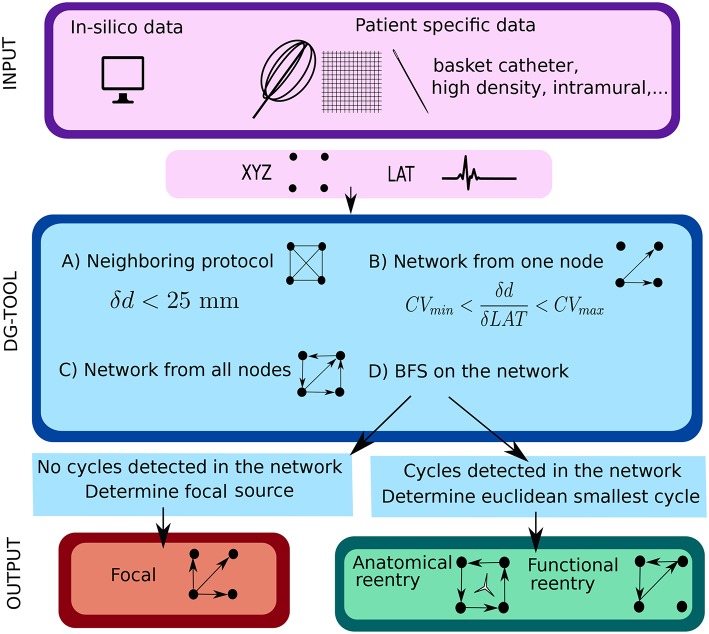
Illustration of the work flow of the DG mapping tool. As input, the DG-tool requires for a given setup of electrodes the LAT values with the corresponding XYZ coordinates, which can be extracted from either simulation studies or a clinical setup. The input is then processed as follows in the DG-tool as presented in the flowchart. Next, we apply a loop-finding algorithm to detect cycles in the network. If cycles are not detected, we locate the source of focal activity. In case cycles were detected, the loops are merged and its center is determined. At the end, the output is visualized. In case of a focal source, arrows pointing away from a (group of) node(s) are shown, while for reentry, arrows will be plotted to visualize the reentry path.

**Figure 2:**
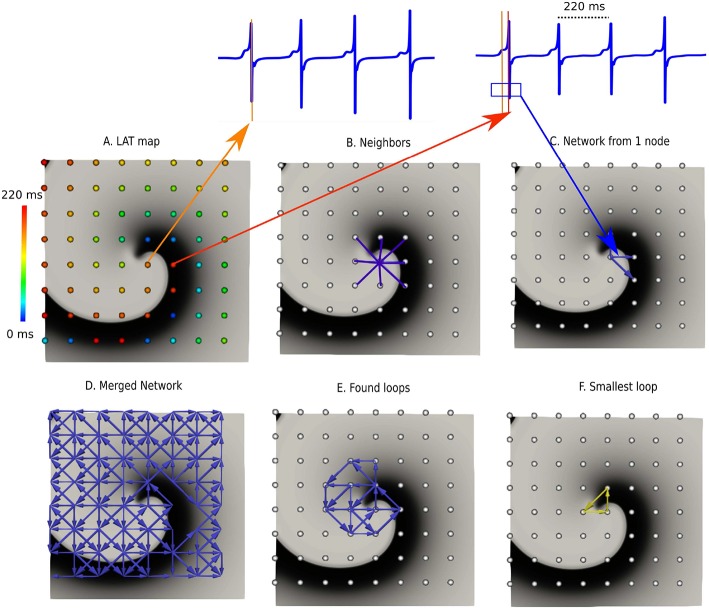
DG mapping for a simulated rotor with a regular measuring grid. **(A)** The points are colored according to their LAT. **(B)** Shows an example of the 8 possible neighbors for a single grid point. **(C)** Represents the corresponding network for the same location. **(D)** The complete network is drawn from which all detected loops in the network are derived, as shown in **(E)**. The selected (smallest) loop among the bundle is shown in **(F)**.

**Figure 3:**
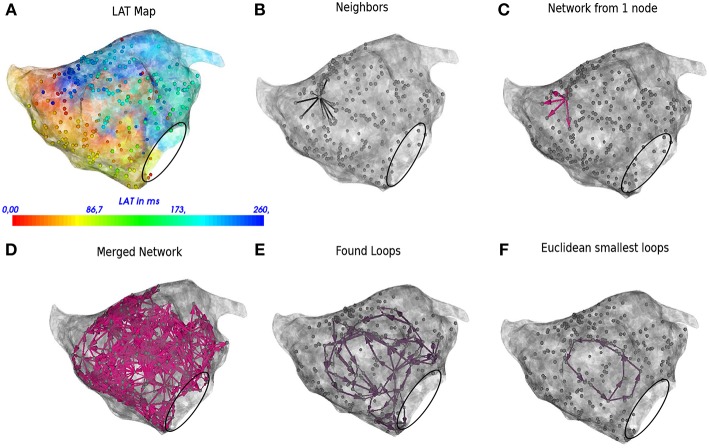
DG mapping for a regular AT case of localized reentry. The gray dots represent the mapping points while the black circle denotes the mitral valve. The LAT annotation map in ms is given in **(A)**. **(B)** Shows an example of all possible neighbors for a single annotated location. **(C)** Represents the corresponding network for the same single annotated location. **(D)** The complete network is drawn from which all detected loops in the network are derived, see **(E)**. The loop corresponding to the smallest cycle in the network among the bundle is shown in **(F)**.

In the next sections the general protocol of DG mapping is explained according to the flowchart given in Figure 1. Figures 2, 3 demonstrate DG mapping on a simulated and a clinical example.

### 2.1. Input

The DG-protocol can be applied to a wide range of *in silico*, experimental and clinical models of arrhythmia with different types of electrode systems (e.g., basket electrode system, intramural needle electrodes, high density grid data, unstructured electrodes systems, etc.). In the current study, this will be demonstrated with *in-silico* generated and clinical data.

#### 2.1.1. *In-silico* Generated Datasets

All simulations were performed using the TNNP-computer model for human ventricular cells (ten Tusscher and Panfilov, 2006) utilizing the explicit-Euler integration scheme (Vandersickel et al., 2014) on a GeForce GTX 680 and a GeForce GTX Titan, with single precision. The following different scenarios were simulated: (1) Functional reentry was simulated in 2D (in a domain of 512 by 512 grid points with interspacing of 0.25 mm) and 3D (a simplified model of the human ventricle and an anatomically accurate model of the human ventricle Tusscher et al., 2007). (2) Anatomical reentry was also simulated in 2D and 3D (anatomically accurate model of the human ventricle). In both scenarios, the S1S2-protocol was applied to obtain rotational activity (Tusscher and Panfilov, 2003). (3) Focal activity was simulated in 2D and 3D (anatomical model of the human ventricle) by applying 3 stimuli at 3 different locations of 500 ms each. All simulations were performed for a duration of 20s. In all simulations, the rotors were stable in space and time.

For each different setup, we implemented either 64 surface electrodes (mimicking 64 electrode-basket catheters, Narayan et al., 2012), 256 surface electrodes with an interspacing of 0.8 mm (mimicking experimental grid sizes, de Groot et al., 2010) or 500 intramural electrodes (in the 3D anatomical model) in analogy with the experimental setup by Taccardi et al. (2005) In Figure 2, an example of a rotor with 64 electrodes is shown. For these electrodes, we computed local unipolar electrogram as follows:

(1) $$\begin{array}{r} ECG\left(t,\vec{x}\right)=\frac{\int \vec{\nabla }V\left(t\right).\left(\vec{r}-\vec{x}\right)}{|\vec{r}-\vec{x}{|}^{3}}d\vec{r} \end{array}$$

where *t* is time, *x* is the distance to the tissue, *V* is the transmembrane potential and *r* is the integration parameter over the tissue. The XYZ-coordinates of the selected electrodes were also stored for further analysis. The LAT of each electrode was determined by taking the steepest negative slope (−*dV*/*dt*) of the calculated unipolar electrogram, see also Figure 2A. This coincides with the upstroke of the action potential (i.e., the true moment of activation) (Spach et al., 1979; Spach and Dolber, 1986).

#### 2.1.2. Clinical Datasets

Between April and August 2017, 29 patients undergoing ablation of symptomatic ATs at AZ Sint-Jan Bruges Hospital were enrolled in the study, resulting in 31 activation maps (30 left atrium, 1 right atrium). The study was approved by the local ethics committee of AZ Sint-Jan Hospital Bruges. High density (>300 points) endocardial mapping of ATs was performed using a single-electrode mapping and ablation catheter with a distal 3.5 mm tip and three 1 mm ring electrodes (THERMOCOOL SMARTTOUCH Biosense-Webster Inc., Diamond Bar, CA, USA). These high density maps covered the full atrium. The bipole of a decapolar coronary sinus (CS) catheter was selected as reference for activation mapping (i.e., peak of CS = 0 ms). The following settings for activation mapping were applied: mapping window set to tachycardia cycle length minus 10 ms and centered at the 0 ms reference. Usually the activation map window is set in this way with the aim to cover the entire tachycardia cycle length during mapping (Del Carpio Munoz et al., 2010). This mapping window is a filter criterion used during continuous mapping that compares LATs between two consecutive beats, but only if the difference in LAT does not exceed 10 ms are the data then acquired. This filter enables correct and accurate data acquisition in order to make a consistent activation map. The other settings were minimum contact force of 4 g, LAT stability of 10 ms, respiratory gating enabled and color fill calibrated at 5. Bipolar scar threshold was defined at 0.05 mV (Anter et al., 2015), and EGMs with bipolar voltages lower than this cutoff were therefore automatically tagged as scarring (gray zones) on the activation maps.

Automated and continuous acquisition of points was performed by the CONFIDENSE mapping module (Carto 3 v. 4, Biosense Webster Inc.) using the novel hybrid LAT annotation method (LATHybrid) (Pooter et al., 2018). Each AT case was analyzed offline by DG mapping after exporting all local activation times (LATs) and the corresponding 3D coordinates. In Figure 3A, an example of the left atrium is shown, with the corresponding LAT map and annotated points.

The tachycardia mechanism was confirmed when ablation resulted in sinus rhythm or in conversion of a second tachycardia. In case of multiple hypotheses of the AT mechanism, the hypothesis which agreed with the ablation endpoint was chosen.

### 2.2. Directed Graph Mapping Protocol

This section explains the DG mapping algorithms, as shown in the blue panels in Figure 1.

#### 2.2.1. Determine the Neighbors in a Given System

First, for a given configuration of electrodes, possible neighbors for each electrode are determined. These neighbors cover all possible paths where the wave can travel to, starting from a certain electrode. For regular grids, the neighbors are found by setting a spheric distance around a single point. Hence, a single point incorporates up to 8 neighbors in case of the 2D grid (see Figure 2B) and up to 26 neighbors in case of a regular 3D grid. For an irregular configuration of electrodes, like the clinical AT cases, Delanauy triangulation is applied to determine for each electrode its possible neighbors (see Figure 3B).

#### 2.2.2. Creating Network of Cardiac Excitation

We chose a certain time *t*. Starting from this time, we find *LAT*_1_, …*LAT*_*n*_ which are the first LAT larger than *t* for each electrode in our system of *n* electrodes. We then draw arrows as follows. Suppose electrodes 1 and 2 form a pair of neighbors. Assume electrode 1 has *LAT*_1_ and electrode 2 has *LAT*_2_, with *LAT*_2_ > *LAT*_1_, meaning the difference between the two electrodes is δ*LAT* = *LAT*_2_ − *LAT*_1_ > 0. We allowed a directed link from electrode 1 to 2 if (Vandersickel et al., 2017):

(2) $$\begin{array}{r} CV_{min}<\frac{\delta d}{\delta LAT}<CV_{max}. \end{array}$$

In this equation, *CV*_*min*_, *CV*_*max*_, and *d* represent minimal conduction velocity, maximal conduction velocity and the euclidean distance between the two electrodes, respectively. For the simulated examples for ventricular tissue (2D and 3D) we took *CV*_*min*_ = 0.2 mm/ms, and *CV*_*max*_ = 2.00 mm/ms. For the clinical AT cases, *CV*_*min*_ was set at 0.08 mm/ms, according to the lowest physiological conduction velocity in human atria determined by Konings et al. (1994), *CV*_*max*_ was set to maximal 2.0 mm/ms (Harrild and Henriquez, 2000). In Figures 2C, 3C, the directed arrows from a single electrode are shown.

Once this first graph was created, a second graph at a time *t* + δ*t* was created in exactly the same way as the first graph. We set δ*t* = 40 ms. Finally, these two graphs were merged, whereby arrows of the second network were added to the first network if the LAT of the node where the arrow originates from was the same. This was necessary as in the first network, no closed cycles will be present, which represent the rotational activity of the arrhythmia, and they are exactly the arrows of the second graph, which will create cycles in the network. The resulting graph is the final directed network. δ*t* = 40 ms is an arbitrarily chosen value to make sure the network indeed forms closed loops, but the algorithm can work as well for other values of 0 < δ*t* < *CL*/2. However, one cannot make δ*t* too small, as otherwise the first and second graph might be equal. For example, in Figures 2D, 3D, the complete network is shown for a simulated case and a regular AT.

#### 2.2.3. Rotational Activity

Once the network is created, any type of rotational activity can be found by detecting cycles in the network. A cycle is a closed directed walk without repetition of nodes. In order to find the cycles, a standard breadth-first search algorithm was used. Since the constructed network generally turns out to be rather small and very sparse, this can be done very efficiently. It turns out that detecting all (smallest) cycles through each node can be done almost instantaneously. We ran theoretical simulations on networks with 1,000,000 nodes, and even in these cases all cycles were found in the range of seconds. Clearly, the physical bounds on the number of electrodes that can be placed will be more limiting than the computational work that is needed to process the data. In Figures 2E, 3E, the resulting cycles of the network of a simulated rotor and a regular AT case are shown.

In order to find the core of any type of rotational activity, we looked for the smallest cycles in the network and computed the geometric center. This was performed by grouping all found cycles based on their proximity to the geometric center. If the centers lie closer to each other than a specified threshold, the cycles were considered to belong to the same core. In this study, we took 1 cm as threshold, as we estimated that the cores of the reentry loops considered in this work were always apart > 1 cm. Afterwards there was an optional pass which merges bundles of cycles if they shared nodes. Finally, the centers of each bundle were defined as the core of rotational activity. In Figures 2F, 3F, the selected cycles are shown.

#### 2.2.4. Focal Activity

Focal activity was detected as a source node, i.e., a node which has a non-zero out-degree, and an in-degree equal to 0. These can be found immediately by doing a single pass over all nodes. Then, the LATs were bundled in certain intervals to reduce the inter-variability in the LAT values. Afterwards, we reconstructed the network with these bundled values. We then checked if regions with only outgoing arrows were present. The middle of these regions corresponds to the source of the focal activity. In Figure S1, we have repeated all the previous steps for a simulated focal source.

### 2.3. Additional Features of Network Theory

In addition to finding rotational and focal activity, we derived additional properties of the network.

#### 2.3.1. Region of Influence

For each network containing reentrant circuits, we can determine a “region of cycles” and a “region of influence.” The region of cycles contains all nodes (electrodes) which are part of cycles for a particular reentrant circuit. Second, for each non-marked point we can determine the closest “region of cycles” in terms of network arrival time distance and relate it to that region. As a result, for each point we can determine which source excited it. This is called the “region of influence.”

In order to construct the region of influence, the following algorithm was implemented. For a given network, all *n* cores were determined, *c*_1_, …, *c*_*n*_. For each core, we first determined all nodes which are part of cycles of the network (*C*_1_, …, *C*_*N*_), i.e., the regions of cycles. Then, each node was added to the core *c*_*i*_ to which it had the shortest path to one of its nodes in *C*_*i*_. In this way, each core is assigned a region of influence.

#### 2.3.2. Wave Averaging

Another application of the constructed network is wave averaging to interpret the cardiac excitation pattern. In general, the outgoing arrows of each node were averaged, and only this average arrow was kept for the visualization. In more detail, the following steps were taken in the wave averaging algorithm. First, each LAT-node was projected on the geometry (mesh) of the atrium. Second, each arrow of the directed network was projected by dividing the arrow in 4 equal parts and projecting these parts on the geometry. The begin and endpoints of these arrows form new nodes which were added to the existing nodes. Then, for each node *n* on the geometry, each directed arrow starting from this node as well as each connection of each node within 1 cm from the original node *n* was averaged. The collection of these averages was then plotted on the geometry.

### 2.4. Phase Mapping Protocol

LAT values were used to construct the excitation patterns in phase-space. First, a sawtooth wave, with amplitude ranging from −π to π is constructed based on these LAT values. Afterwards, values are adapted with their 2π equivalent within the range of −π to π in phase-space. Next, in both x and y directions, the phases were derived and a linear combination with the Sobel (we also tested the Nabla) kernels to detect the singularities was applied. This protocol was previously presented (Bray et al., 2001; Bray and Wikswo, 2002a,b; Umapathy et al., 2010; Tomii et al., 2016). However, based on the properties of the ECGs of the simulation, we made use of this sawtooth wave instead of the more regular Hilbert transform of the ECG signal, as this is does not make any difference for regular signals—see Kuklik et al. (2015). In 3D, the heart was sliced in 3 orthogonal directions and the protocol was applied on each slice. However, as the shape of the ventricle model is complex, only grid points with complete circumference in the heart were taken into account, so convolution did not result in false positives on the edges (Van Nieuwenhuyse et al., 2017). However, this did not result in detection of the filament of the rotor as the density (500 intramural points) was too sparse. We therefore calculated the phase singularities on the surface of the tissue and detected eventually the phase singularities of the spiral in 3D. A binary detection threshold was applied to the convolution (Tomii et al., 2016), set to 95% of the maximal detected value in phase-space.

### 2.5. Introducing LAT Variation

In the clinical setup, identification of LAT either by automated algorithms or manual annotation by operators can vary due to several factors such as accuracy of the detection algorithms, operator experience, signal quality and noise (El Haddad et al., 2013). Therefore, we included LAT variation in our analysis, and compared the accuracy of DG mapping with phase mapping. In order to obtain LAT variation in the simulations of functional reentry, random Gaussian noise was added with standard deviations σ = 5, 10, 15, 20, 25, 30 ms in the simulation of functional reentry with a configuration of 64 and 256 electrodes. We divided the activation times obtained during a simulation in 25 different frames with 520 ms separation to exclude any overlap in activation times. For each frame, we randomly added Gaussian noise 1,000 times, so in total, we compared 25,000 different frames per LAT variation σ. The center of the rotor was detected through DG mapping and phase mapping. For DG mapping, the geometric center of all cycles belonging to the same core was computed. Afterwards, the median value was taken as the true center of the rotor. In addition, only the center with the highest number of cycles was taken into account. We classified the outcome as correct if only one single core was found within 1 cm of the true core. The incorrect diagnosis was classified in 3 different types: incorrect cores (i.e., cores outside a 1 cm radius of the true core) in combination with the correct core (error type 1), only incorrect cores (error type 2), or no cores (error type 3). For the percentage correct diagnosis *p*, we computed a 95% confidence interval via *p* ± 1.96*SE* where *SE* is obtained from a robust sandwich estimator (Thomas Lumley, 2015; R Core Team, 2017) that accounts for the correlation structure (i.e., the 1,000 replicates within one time frame are expected to be correlated). In the Supplementary Material, we also simulated noise from the skewed lognormal distribution to study the robustness of the methods for different types of noise distributions. In addition, we also presented the outcome as a function of the distance from the true core (instead of taking 1.0 cm).

## 3. Results

### 3.1. *In-silico* Models of Functional and Anatomical Reentry and Focal Activity

**Figure 4:**
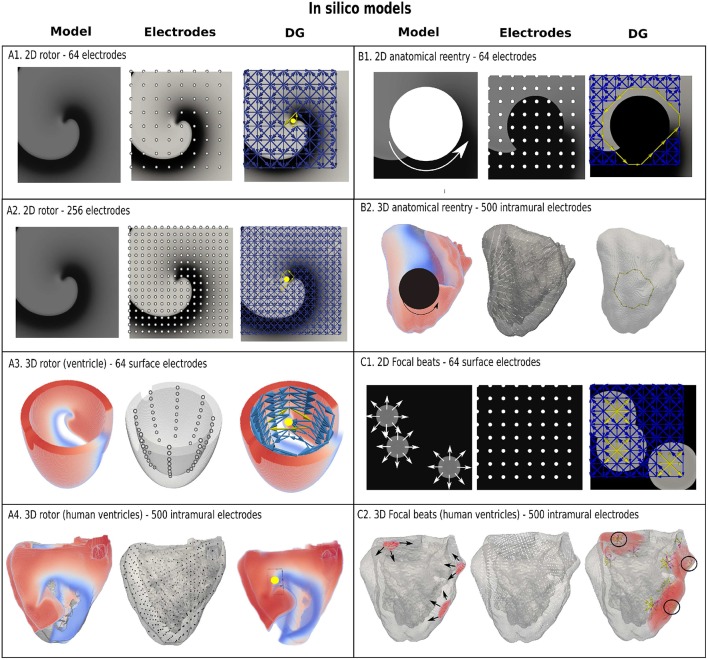
Examples of DG mapping for different *in-silico* models. Model **(A1,A2)** show a rotor in 2D with 64 and 256 electrodes. Model **(A3)** shows a rotor in a simplified model of the ventricle with 64 surface electrodes. Model **(A4)** depicts a rotor in an anatomical model of the ventricles with 500 intramural electrodes. The smallest cycles and their geometrical center are indicated in yellow. Model **(B1)** depicts anatomical reentry in 2D with 64 electrodes and Model **(B2)** in a model of the human ventricles with 500 intramural electrodes. The smallest reentry loop around the obstacle is again depicted in yellow. Model **(C1)** represents 3 focal sources in 2D with 64 electrodes, whereby DG mapping shows the sources in yellow. Similar results with 3 focal beats in the ventricles are shown in model **(C2)**, indicated with 3 black circles. In addition, the edges of the moving wavefronts are also found by DG mapping, see model **(C2)**, which depend on the time the graph is constructed.

The accuracy of DG mapping was tested in different *in-silico* models as described in the methods section. First, for functional reentry (see Figure 4A), we simulated a 2D rotor with a configuration of 64 electrodes (A1) and 256 electrodes (A2). In 3D, functional reentry was induced in a simplified model of the ventricles with 64 surface electrodes (A3) and in an anatomical model of the ventricles with 500 intramural electrodes (A4). In all four setups, DG mapping was able to accurately detect functional reentry and correctly determine the location of the core of the rotor for the entire length of the simulation (20 s duration). The smallest cycle and corresponding core are shown in yellow for each setup. Second, DG mapping was validated in two models of anatomical reentry (Figure 4B): a 2D anatomical circuit with 64 electrodes (B1) and a 3D anatomical reentry with 500 intramural points in the model of the ventricles (B2). In both models, DG mapping correctly identified the reentrant path around the obstacles for the entire length of the simulation (20s). The shortest reentry loops are again depicted in yellow. Third, focal activity was simulated in 2D (64 electrodes) and 3D (500 intramural electrodes) (Figure 4C) by repetitively stimulating 3 different locations. Again, DG mapping identified the electrodes most closely to the site of stimulation, see C1 (yellow arrows) and C2 (black circles).

### 3.2. Clinical Dataset

**Figure 5:**
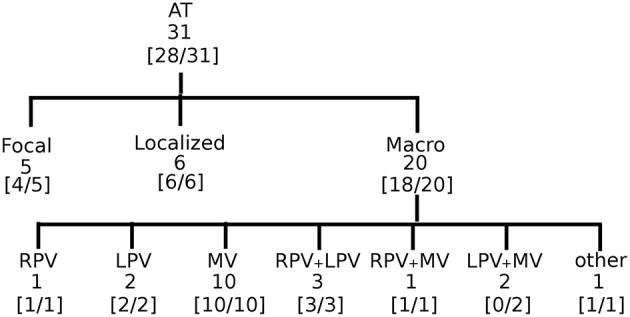
Accuracy of DG mapping in clinical AT. The gold standard was compatible with focal source (5), localized reentry (6), and macro-reentry (20). Macro reentry was categorized in reentry around the right veins (RV), the left veins (LV), mitral valve (MV), around RV + LV, around RV + MV, around LV + MV, and other types of reentry (e.g., in the right atrium). In brackets the accuracy of DG mapping is given.

To establish proof of concept in the clinical setting, we retrospectively and blindly analyzed 31 cases of regular atrial tachycardia (AT). For clarity, in Figure 3, all the steps of the DG mapping protocol were demonstrated on an AT case of a localized reentry. In general, the atria have a complex structure. In case of reentry during AT, the electrical waves circle around obstacles such as the valves, the veins or scar tissue, creating a (sustained) reentry loop. Ablation aims to terminate the reentry loop so that the circular electrical conduction can no longer be sustained.Therefore, it is important to precisely determine the location of the activation pathway. The accuracy of DG mapping was compared to the standard diagnosis, i.e., type of arrhythmia and location of the circuit/focal activity as determined by the electrophysiologist (EP) based on the activation map and the ablation result. The overall results are summarized in Figure 5. Out of 31 cases, 20 were due to macro-reentry, 6 due to localized reentry and 5 due to focal activity—see also the Table S1. In 9 cases with reentry, the operator was not sure about the reentry mechanism purely based on the LAT activation map, formulating several hypotheses. The gold standard was taken as the diagnosis matching the ablation endpoint. Compared to this gold standard diagnosis, DG mapping identified the exact same mechanism and location in 28 out of 31 cases (90.3%, 95% exact binomial confidence interval 74.2% - 98%). In 3 out of 31 cases, the diagnosis of DG mapping did not fully match with the gold standard. In 2 cases of double loop reentry (cases 6 and 14), DG mapping identified only one single loop. In the other case (case 22), the mapping data indicated focal tachycardia, whereas DG mapping identified localized reentry at the same location. However, in all 3 cases, DG mapping would have pointed to the correct ablation target, meaning that DG mapping correctly identified the ablation target in 31/31 cases.

**Figure 6:**
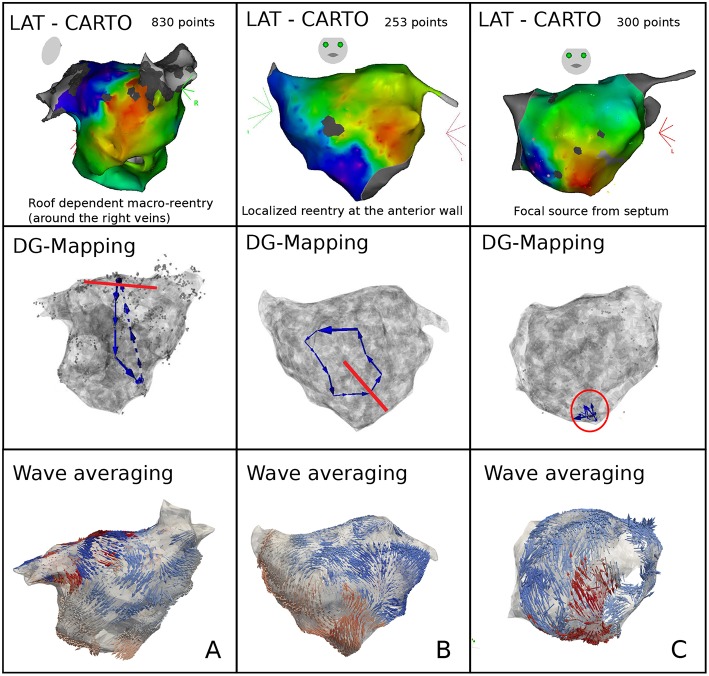
Representative examples of DG mapping in 3 cases of clinical AT: macro-reentry **(A)**, localized reentry **(B)**, and a focal source **(C)**. For each case, the activation map (top), the corresponding DG-map whereby the ablation target is indicated in red (middle) and the network based wave averaging (bottom) are given.

Representative cases are shown in Figure 6. Panel A depicts a macro-reentrant AT around the right pulmonary veins in the LA conducting over the roof. Ablation of the roof resulted in prompt termination of the AT. Blinded analysis by DG mapping revealed a selected loop at the same location (middle panel). Panel B shows a localized reentry at the anterior wall, rotating around local scar tissue. Ablation from the scar to the mitral valve terminated AT. DG mapping (middle) as well as wave averaging (bottom) identified the same location of the localized reentry. In panel C, activation mapping and ablation conformed with focal tachycardia at the septum. DG mapping (in the absence of loops) pointed to focal activity as well (middle panel). We also tested the wave algorithm for each case, as shown in the bottom panels of Figure 6. Wave averaging was for all the cases compatible with the results of the DG mapping. Representative examples are shown in Figure 6: macro reentry (A), localized reentry (B), and focal activation (C).

### 3.3. Comparison With Phase Mapping Under LAT-Variation

**Figure 7:**
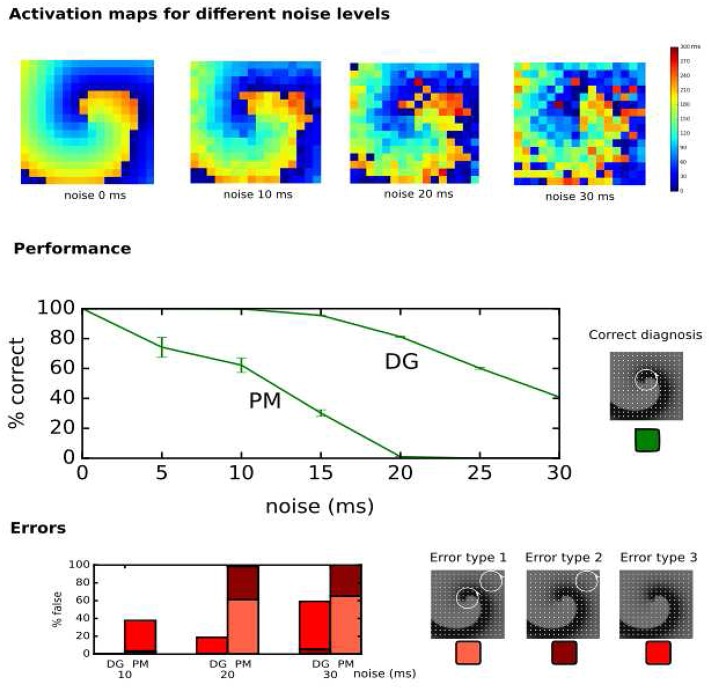
Performance of DG mapping and phase mapping (PM) while adding Gaussian noise with an increasing standard deviation (ranging from 0 to 30 ms) in a single 2D rotor model with 256 electrodes. The upper panels show representative activation maps for different levels of noise. The middle panel shows the performance of DG mapping vs. phase mapping for these different noise levels. The bottom panel shows the type of errors in case of failure for both methods. Error type 1 is a detection of a false core in addition to the correct core, error type 2 is only a detection of a false core and error type 3 is no detection of cores at all.

In the clinical setup, LATs can vary due to several factors such as accuracy of the detection algorithms, operator experience, signal quality and noise (El Haddad et al., 2013). Therefore, the performance of DG mapping was compared to phase mapping in the model of a single rotor with 256 electrodes, now by adding Gaussian white noise to the LATs (Figure 7, upper panels). Overall, we observed that DG mapping retains its accuracy to detect rotors at increasing noise levels, whereas phase mapping becomes less precise (middle panel of Figure 7): for small variation levels (5 ms), DG mapping is 100% accurate, while the accuracy of phase mapping decreased to 74.17%. For 15 ms, phase mapping became highly unreliable (accuracy of 30.22%) while DG mapping had an accuracy of 95.49%. For 20 ms, this difference was even more pronounced: DG mapping maintained an accuracy of 81.19% while the accuracy of phase mapping dropped to 1.08% (*p*-value < 0.0001). Moreover, in case of incorrect diagnosis (lower panels), phase mapping detected extra false cores (type 1 error) whereas in the DG method incorrect diagnosis was due to no detection of the core (type 3 error). Noise analysis was repeated for the 2D model with 64 electrodes. A similar trend was found with DG mapping being more accurate (91%, 83%, 68%, for a noise level of 10, 15 and 20 ms) vs. phase mapping (75%, 51%, 21%, respectively). All of these differences were highly significant (*p*-value < 0.0001), see also Table S2. We also varied the distance to the true core for which we retained a diagnosis as correct, as shown in Figure S2 (and see also Figure S3 for more explanation). As explained in the supplementary material, due to the discrete nature of phase mapping, we can only compare phase mapping and DG mapping above a certain threshold. In these cases, DG mapping always exceededs phase mapping. We also tested the effect of the underlying distribution of the LAT values. Modeling LAT variation with the skewed lognormal distribution did not alter the conclusions of the results—see also Figure S1. Finally, to test the specificity of DG mapping, we applied DG mapping in a point stimulation model without functional reentry (256 electrodes, LAT variation ranging from 0 ms to 30 ms). In these cases, DG mapping never identified any rotors, resulting in a specificity of 100%.

### 3.4. Region of Influence

**Figure 8:**
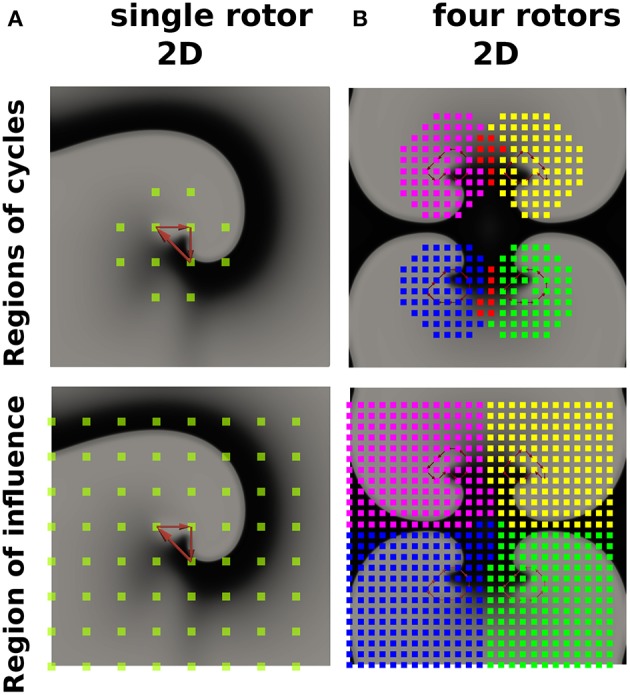
The regions of cycles and the regions of influences for model **(A)** (single rotor) and model **(B)** (four rotors). Models **(A,B)** were simulated with 64 resp. 625 electrodes. The smallest cycles are depicted in red. Different regions of cycles were depicted in different colors, while the overlap is shown in red. Similarly, different regions of influence are shown in different colors, dividing model B into four different regions.

Describing cardiac excitation as a network allows the extraction of additional information. Besides wave averaging, DG mapping allows the identification of the spatial region which is excited by a certain source. In case of normal excitation, a single source (sinus node) excites the whole medium. However, in case of an arrhythmia with multiple sources, each source excites a given region, which we call the region of influence. We hypothesized that DG mapping, by containing complete spatio-temporal information, can determine this area of influence. This concept was evaluated in 2 different setups (see Figure 8A). We determined the region which contained the electrodes belonging to cycles (region of cycles) as well as the region of influence. Obviously, for a single rotor, the region of influence spans the entire set of electrodes. For 4 different rotors, one can observe an area of influence for each given source.

## 4. Discussion

### 4.1. Main Findings

In this paper, we demonstrated that a directed network can be used to describe electrical activity in the heart in order to find the mechanism of the arrhythmia. This novel method is robust, fast, general, accurate and can be applied to a wide range of *in silico*, experimental and clinical models of arrhythmias. First, we showed that DG mapping can find functional reentry, anatomical reentry and focal activity in *in-silico* ventricular models of the heart (see Figure 4). We tested using intramural electrodes, 64-basket electrodes and regular grids with different numbers of electrodes (64-256). Second, we tested DG mapping on 31 clinical cases of regular AT—see Figure 6. Compared to the gold standard, DG mapping identified the exact same mechanism and location in 28 out of 31 cases, whereas it identified the correct ablation target in all 31 cases. These results suggest that DG mapping could potentially lead to improved treatment of tachyarrhythmias based on stable sources.

### 4.2. Network Theory

To our knowledge, so far only limited research has focused on network theory to understand cardiac arrhythmias. In the study by Zahid et al. (2016), undirected networks were used to find the minimal number of nodes which need to be ablated to separate two regions in the heart. This region was then proposed as the optimal ablation site. In the study by Tao et al. (2017), the authors showed that ablation of persistent AF is associated with improvement in both local and global connectivity within the communication networks. However, in neither of the above studies, excitation was interpreted as a *directed* network. Zeemering et al. (2013) applied a directed network to describe AF by accumulating multiple time frames. However, in contrast to DG mapping, this methodology precluded the possibility of detecting rotational activity and does not represent the actual wave excitation. Also in Richter et al. (2012), AF was described as a directed network via the use of sparse modeling for the estimation of propagation patterns in intracardiac atrial fibrillation. However, it is not clear how rotational activity can be detected from the obtained networks, and it would be of interest to uncover the cycles. Similarly, in Alcaine et al. (2016) and Luengo et al. (2018), directed arrows are created based on the concept of Granger causality between different signals instead of the LATs. This could form an alternative way to create the directed network if deriving the LATs from the signals is not feasible. In a model of chronic atrial ventricular block, we used directed networks for the first time to determine the mechanism underlying Torsade de Pointes (Vandersickel et al., 2017). However, again, we did not use it to fully describe the electrical excitation as in this work. Therefore, to our knowledge, this is the first study where directed networks were used to describe electrical excitation to extract the mechanism of the arrhythmia, building on our previous work in the CAVB dog (Vandersickel et al., 2017).

### 4.3. Advantages of DG Mapping

First, we showed that DG mapping could be used to reliably detect rotational activity even after adding LAT variation. For instance, in the model presented in Figure 7, we found that in case of 15 ms standard deviation of noise, phase mapping was only 30% accurate, while DG mapping was still 96% accurate. This difference in accuracy can be explained by the holistic nature of DG mapping. In contrast with phase mapping, in the presence of a number of electrodes with a wrong LAT annotation, DG mapping can still identify the correct location of the rotor based upon the other electrodes. In addition, DG mapping also takes into account the number of cycles which are found for each rotor (with a higher number of loops indicating a higher likelihood of an actual rotation). In contrast, phase mapping finds phase singularities locally. Therefore, a misplaced LAT can easily give rise to false positives/negatives, potentially resulting in incorrect clinical decisions. Also, DG mapping takes into account the conduction velocities of the excitation, which corrects for non-regular spacing of the electrodes, a feature which phase mapping lacks.

Second, DG mapping automatically detects focal activity, a feature which phase mapping lacks. Also, depending on the size of the obstacle and the spacing of the electrodes, phase mapping cannot detect anatomical reentry. In addition, phase mapping might indicate anatomical reentry around a small obstacle as functional reentry. Very recently, another methodology was described by Oesterlein et al. which can also automatically detect anatomical reentry (Oesterlein et al., 2018). The approach uses a different methodology based on integral measures: determination of activated area and its relation to the cycle length of the arrhythmia, while our DG mapping directly analyzes the local propagation of the excitation wave. It would be interesting to compare this method with DG mapping, especially in clinical settings. Also, other techniques exist to help uncover the mechanism for atrial arrhythmias like the ripple map (Linton et al., 2009) (AT) or the retro mapping technique (Mann et al., 2019) (AF). However, both methods still require a manual interpretation of the novel types of maps which were obtained by these techniques. Also very recently, the STAR technique was developed (Honarbakhsh et al., 2019). The STAR methodology aims to identify the predominant wavefront direction by displaying it on the STAR maps. Although it does not focus on identifying a particular arrhythmia mechanism, it helps the operator to determine the mechanism from the STAR maps.

Third, DG mapping offers additional features which can be derived from the directed network. DG mapping can determine all electrodes belonging to any cycle which are part of the same rotational activity (see Figure 8A) and detect for each rotational core its region of influence (Figure 8B). This offers the possibility to detect all electrodes activated by a specific rotational activity and could detect the dominant driving source of the arrhythmia as a primary target of ablation. Also, the wave averaging technique allows the creation of maps of the wave propagation, which can provide additional guidance during catheter ablation.

Finally, another advantage is that DG mapping is universal. It can be applied to any type of recording system from which LATs can be derived, with varying number of electrodes, the inter-electrode distance or site of recording, as shown in Figure 4.

### 4.4. Clinical Implications

As shown in this paper, DG mapping can be of added value in the ablation of regular AT. Despite improvements in activation mapping (RHYTHMIA by Boston Scientific, Coherent mapping system by Biosense Webster), interpretation of activation maps remains challenging and operator dependent (Gerstenfeld and Marchlinski, 2007; Kaiser et al., 2018). We demonstrated that DG mapping automatically identified the same mechanism as the electrophysiologist (EP) in 28/31 cases of regular AT, but did find the correct ablation target in 31/31 cases. Moreover, in 9 cases with reentry, the operator was not sure about the mechanism based on the LAT activation map and formulated several hypotheses (see Table S1). Therefore, DG mapping could aid physicians in finding the correct diagnosis according to the ablation target. Currently, in case of doubt, the operator can perform entrainment mapping whereby the post pacing interval (PPI) is compared to the tachycardia cycle length (TCL) to localize or confirm the correct reentry circuit (Knight et al., 1999). Furthermore, DG mapping also automatically detects focal activity and its location, making it a complete diagnostic tool for AT. Compared to the standard assessments, DG mapping is robust, fast and operator independent. Therefore, DG mapping would remove the manual interpretation of the (experienced) operator. The wave averaging technique allows the creation of maps of the wave propagation, which can provide additional guidance during catheter ablation. Another advantage is that DG mapping is instantaneous and could therefore shorten the ablation procedure.

Another important potential application could be AF. AF is often referred to as the most common arrhythmia in clinical practice, with an estimated prevalence of 2%, and is associated with a fivefold and twofold higher risk of stroke and death, respectively (Zoni-Berisso et al., 2014). Catheter ablation of AF yields moderate success rates (Brooks et al., 2010; Weerasooriya et al., 2011; Weiss et al., 2016), which is related to the lack of understanding of AF mechanisms. Different mechanisms for AF have been described such as focal activation, dissociated activity or stable rotors (Allessie and de Groot, 2014a,b; Narayan and Jalife, 2014a,b; Chen et al., 2015; Kirchhof et al., 2016). Currently, both researchers and electrophysiologists rely on activation mapping or phase mapping for the analysis of AF. Recently, initial studies suggested good outcomes after ablation of rotors guided by phase mapping (Narayan et al., 2012, 2014). However, new studies have emerged contradicting this study (Buch et al., 2016; Gianni et al., 2016; Mohanty et al., 2016). It was shown that phase mapping easily generates false positives (Vijayakumar et al., 2016; Kuklik et al., 2017), especially due to LAT variations on the signals and large inter-electrode distances. In our study, phase mapping showed similar results when adding LAT variations, whereas DG mapping maintained a high accuracy. It remains to be seen whether DG mapping will offer new insight in AF mechanisms; however, the holistic nature of the method (as explained in the advantages of DG mapping) might overcome the problems with phase mapping, as currently used in the clinic.

In conclusion, translating cardiac arrhythmias into directed networks as described in the current work offers the beginning of a new area of research. There exists a whole range of different algorithms in network theory (e.g., edge density, centrality measures, etc. White and Borgatti, 1994; Newman, 2010; Holme, 2015; Sizemore et al., 2018), which can possibly be applied to the constructed networks to increase our understanding of cardiac arrhythmias.

### 4.5. Limitations

As this paper was a proof-of-concept, many different settings are not yet tested. For example, it remains to be tested how DG mapping will characterize cardiac excitation in more complicated settings with multiple meandering rotors, including wavebreaks, or in complex fibrotic tissue. A limitation of DG mapping is that it requires at least one full cycle (as DG mapping can only find closed loops) of a circular rotation, while phase mapping finds phase singularities instantaneously. A limitation of DG mapping is that it requires a degree of stability of the cardiac excitation pattern since a full cycle of activation is required for DG mapping. Therefore it remains to be seen in the clinical setting if DG mapping can advance the understanding of more complex arrhythmia such as AF, VT and VF. For these cases, DG mapping requires the arrhythmia to be mapped first, which is not always possible (e.g., due to hemodynamic instability in VT/VF etc.). Future studies are needed to further evaluate DG mapping in different types of arrhythmias.

## Data Availability

The datasets generated for this study are available on request to the corresponding author.

## Ethics Statement

The studies involving human participants were reviewed and approved by ethics committee of AZ Sint-Jan Hospital Bruges. The patients/participants provided their written informed consent to participate in this study.

## Author Contributions

NV coordinated the project, set up the collaboration, performed the computer simulations, and developed the DG-mapping protocol. EV developed and optimized software for the study of DG-mapping in simulations, all clinical AT cases, and analyzed simulations with phase mapping. JG and NVC developed the software to analyze the networks created by NV and EV. ME provided all data and analyzed the clinical data. JD performed the statistical analyses. TS contributed in analyzing the clinical cases. AD contributed in writing the paper. MD analyzed all 31 clinical cases, performed all ablations which were analyzed in this work, and significantly contributed to all parts of the paper. AP contributed to all parts of the paper and co-developed a part of the protocol (region of influence). All authors contributed and collaborated in writing and improving all aspects of the paper.

### Conflict of Interest Statement

The authors declare that the research was conducted in the absence of any commercial or financial relationships that could be construed as a potential conflict of interest.

## References

[B1] Alcaine A. Mase M. Cristoforetti A. Ravelli F. Nollo G. Laguna P.. (2016). A multi-variate predictability framework to assess invasive cardiac activity and interactions during atrial fibrillation. IEEE Trans. Biomed. Eng. 64, 1157–1168. 10.1109/TBME.2016.2592953 27448337

[B2] Allessie M. de Groot N. (2014a). Crosstalk opposing view: rotors have not been demonstrated to be the drivers of atrial fibrillation. J. Physiol. 592, 3167–3170. 10.1113/jphysiol.2014.271809 25085969 PMC4146363

[B3] Allessie M. de Groot N. (2014b). Rebuttal from Maurits Allessie and Natasja de Groot. J. Physiol. 592:3173. 10.1113/jphysiol.2014.275404 25085971 PMC4146365

[B4] Anter E. Tschabrunn C. M. Josephson M. E. (2015). High-resolution mapping of scar-related atrial arrhythmias using smaller electrodes with closer interelectrode spacing. Circulation 8, 537–545. 10.1161/CIRCEP.114.002737 25792508

[B5] Barabási A.-L. (2016). Network Science. Cambridge: Cambridge University Press.

[B6] Barabási A.-L. Gulbahce N. Loscalzo J. (2011). Network medicine: a network-based approach to human disease. Nat. Rev. Genet. 12, 56–68. 10.1038/nrg2918 21164525 PMC3140052

[B7] Barabasi A.-L. Oltvai Z. N. (2004). Network biology: understanding the cell's functional organization. Nat. Rev. Genet. 5, 101–113. 10.1038/nrg1272 14735121

[B8] Borgatti S. P. Mehra A. Brass D. J. Labianca G. (2009). Network analysis in the social sciences. Science 323, 892–895. 10.1126/science.1165821 19213908

[B9] Bray M.-A. Wikswo J. P. (2002a). Considerations in phase plane analysis for nonstationary reentrant cardiac behavior. Phys. Rev. E 65:051902. 10.1103/PhysRevE.65.051902 12059588

[B10] Bray M. A. Lin S. F. Aliev R. R. Roth B. J. Wikswo J. P. (2001). Experimental and theoretical analysis of phase singularity dynamics in cardiac tissue. J. Cardiovasc. Electrophysiol. 12, 716–722. 10.1046/j.1540-8167.2001.00716.x 11405407

[B11] Bray M. A. Wikswo J. P. (2002b). Use of topological charge to determine filament location and dynamics in a numerical model of scroll wave activity. IEEE Trans. Biomed. Eng. 49, 1086–1093. 10.1109/TBME.2002.803516 12374332

[B12] Brin S. Page L. (2012). Reprint of: the anatomy of a large-scale hypertextual web search engine. Comput. Netw. 56, 3825–3833. 10.1016/j.comnet.2012.10.007

[B13] Brockmann D. Helbing D. (2013). The hidden geometry of complex, network-driven contagion phenomena. Science 342, 1337–1342. 10.1126/science.1245200 24337289

[B14] Brooks A. G. Stiles M. K. Laborderie J. Lau D. H. Kuklik P. Shipp N. J.. (2010). Outcomes of long-standing persistent atrial fibrillation ablation: a systematic review. Heart Rhythm 7, 835–846. 10.1016/j.hrthm.2010.01.017 20206320

[B15] Buch E. Share M. Tung R. Benharash P. Sharma P. Koneru J.. (2016). Long-term clinical outcomes of focal impulse and rotor modulation for treatment of atrial fibrillation: a multicenter experience. Heart Rhythm 13, 636–641. 10.1016/j.hrthm.2015.10.031 26498260 PMC4762742

[B16] Chen S.-A. Ravelli F. Mase M. Bollmann A. Kosiuk J. Hindricks G. (2015). Comments on the crosstalk proposal and opposing view: rotors have/have not been demonstrated to drive human atrial fibrillation. J. Physiol. 592(Pt 15), 3163–3166.

[B17] Chugh A. Oral H. Lemola K. Hall B. Cheung P. Good E.. (2005). Prevalence, mechanisms, and clinical significance of macroreentrant atrial tachycardia during and following left atrial ablation for atrial fibrillation. Heart Rhythm 2, 464–471. 10.1016/j.hrthm.2005.01.027 15840468

[B18] Danon L. Ford A. P. House T. Jewell C. P. Keeling M. J. Roberts G. O.. (2011). Networks and the epidemiology of infectious disease. Interdiscip. Perspect. Infect. Dis. 2011:284909. 10.1155/2011/284909 21437001 PMC3062985

[B19] de Groot N. M. Houben R. P. Smeets J. L. Boersma E. Schotten U. Schalij M. J. (2010). Electropathological substrate of longstanding persistent atrial fibrillation in patients with structural heart diseaseclinical perspective. Circulation 122, 1674–1682. 10.1161/CIRCULATIONAHA.109.910901 20937979

[B20] De Pooter J. El Haddad M. Wolf M. Phlips T. Van Heuverswyn F. Timmers L.. (2018). Clinical assessment and comparison of annotation algorithms in high-density mapping of regular atrial tachycardias. J. Cardiovasc. Electrophysiol. 29, 177–185. 10.1111/jce.13371 29059485

[B21] Deisenhofer I. Estner H. Zrenner B. Schreieck J. Weyerbrock S. Hessling G.. (2006). Left atrial tachycardia after circumferential pulmonary vein ablation for atrial fibrillation: incidence, electrophysiological characteristics, and results of radiofrequency ablation. Europace 8, 573–582. 10.1093/europace/eul077 16864612

[B22] Del Carpio Munoz F. Buescher T. L. Asirvatham S. J. (2010). Three-dimensional mapping of cardiac arrhythmias: what do the colors really mean? Circulation 3, e6–e11. 10.1161/CIRCEP.110.960161 21156773

[B23] Delacretaz E. Ganz L. I. Soejima K. Friedman P. L. Walsh E. P. Triedman J. K. (2001). Multiple atrial macro–re-entry circuits in adults with repaired congenital heart disease: entrainment mapping combined with three-dimensional electroanatomic mapping. J. Am. Coll. Cardiol. 37, 1665–1676. 10.1016/S0735-1097(01)01192-5 11345382

[B24] El Haddad M. Houben R. Stroobandt R. Van Heuverswyn F. Tavernier R. Duytschaever M. (2013). Algorithmic detection of the beginning and end of bipolar electrograms: implications for novel methods to assess local activation time during atrial tachycardia. Biomed. Sig. Process. Cont. 8, 981–991. 10.1016/j.bspc.2012.11.005

[B25] Gerstenfeld E. P. Marchlinski F. E. (2007). Mapping and ablation of left atrial tachycardias occurring after atrial fibrillation ablation. Heart Rhythm 4, S65–S72. 10.1016/j.hrthm.2007.01.024 17336888

[B26] Gianni C. Mohanty S. Di Biase L. Metz T. Trivedi C. Gökoglan Y.. (2016). Acute and early outcomes of focal impulse and rotor modulation (firm)-guided rotors-only ablation in patients with nonparoxysmal atrial fibrillation. Heart Rhythm 13, 830–835. 10.1016/j.hrthm.2015.12.028 26706193

[B27] Gray R. A. Pertsov A. M. Jalife J. (1998). Spatial and temporal organization during cardiac fibrillation. Nature 392:75. 10.1038/32164 9510249

[B28] Harrild D. Henriquez C. (2000). A computer model of normal conduction in the human atria. Circul. Res. 87, e25–e36. 10.1161/01.RES.87.7.e25 11009627

[B29] Holme P. (2015). Modern temporal network theory: a colloquium. Eur. Phys. J. B 88, 1–30. 10.1140/epjb/e2015-60657-4

[B30] Honarbakhsh S. Hunter R. J. Finlay M. Ullah W. Keating E. Tinker A. (2019). Development, *in vitro* validation and human application of a novel method to identify arrhythmia mechanisms: the stochastic trajectory analysis of ranked signals mapping method. J. Cardiovasc. Electrophysiol. 9:e004409 10.1111/jce.13882 PMC8609431 30801836

[B31] JaÏs P. Sanders P. HSU L.-F. Hocini M. Sacher F. Takahashi Y.. (2006). Flutter localized to the anterior left atrium after catheter ablation of atrial fibrillation. J. Cardiovasc. Electrophysiol. 17, 279–285. 10.1111/j.1540-8167.2005.00292.x 16643401

[B32] Kaiser D. W. Rogers A. J. Narayan S. M. (2018). Predictability in complex atrial arrhythmias: the n/n–1 algorithm to guide ablation of atrial tachycardias. Heart Rhythm 16, 562–563. 10.1016/j.hrthm.2018.11.018 30465903 PMC6467220

[B33] Kirchhof P. Benussi S. Kotecha D. Ahlsson A. Atar D. Casadei B.. (2016). 2016 esc guidelines for the management of atrial fibrillation developed in collaboration with eacts. Europace 18, 1609–1678. 10.1093/europace/euw295 27567465

[B34] Knight B. P. Zivin A. Souza J. Flemming M. Pelosi F. Goyal R.. (1999). A technique for the rapid diagnosis of atrial tachycardia in the electrophysiology laboratory. J. Am. Coll. Cardiol. 33, 775–781. 10.1016/S0735-1097(98)00614-7 10080480

[B35] Konings K. T. Kirchhof C. J. Smeets J. R. Wellens H. J. Penn O. C. Allessie M. A. (1994). High-density mapping of electrically induced atrial fibrillation in humans. Circulation 89, 1665–1680. 10.1161/01.CIR.89.4.1665 8149534

[B36] Kroto H. Heath J. O'Brien S. Curl R. Smalley R. (1985). *C*_60_: buckminsterfullerene. Nature 318, 162–163. 10.1038/318162a0

[B37] Kuklik P. Zeemering S. Maesen B. Maessen J. Crijns H. J. Verheule S.. (2015). Reconstruction of instantaneous phase of unipolar atrial contact electrogram using a concept of sinusoidal recomposition and hilbert transform. IEEE Trans. Biomed. Eng. 62, 296–302. 10.1109/TBME.2014.2350029 25148659

[B38] Kuklik P. Zeemering S. van Hunnik A. Maesen B. Pison L. Lau D. H.. (2017). Identification of rotors during human atrial fibrillation using contact mapping and phase singularity detection: technical considerations. IEEE Trans. Biomed. Eng. 64, 310–318. 10.1109/TBME.2016.2554660 27101596

[B39] Linton N. W. Koa-Wing M. Francis D. P. Kojodjojo P. Lim P. B. Salukhe T. V.. (2009). Cardiac ripple mapping: a novel three-dimensional visualization method for use with electroanatomic mapping of cardiac arrhythmias. Heart Rhythm 6, 1754–1762. 10.1016/j.hrthm.2009.08.038 19959125

[B40] Luengo D. Rios-Munoz G. Elvira V. Sanchez C. Artes-Rodriguez A. (2018). Hierarchical algorithms for causality retrieval in atrial fibrillation intracavitary electrograms. IEEE J. Biomed. Health Informat. 23, 143–155. 10.1109/JBHI.2018.2805773 29994646

[B41] Mann I. Coyle C. Qureshi N. Nagy S. Z. Koa-Wing M. Lim P. B.. (2019). Evaluation of a new algorithm for tracking activation during atrial fibrillation using multipolar catheters in humans. J. Cardiovasc. Electrophysiol. 10.1111/jce.14033 31211473

[B42] Martin R. Maury P. Bisceglia C. Wong T. Estner H. Meyer C. (2018). Characteristics of scar-related ventricular tachycardia circuits using ultra-high-density mapping: a multi-center study. Circulation 11:e006569 10.1161/CIRCEP.118.006569 30354406

[B43] Mohanty S. Gianni C. Mohanty P. Halbfass P. Metz T. Trivedi C.. (2016). Impact of rotor ablation in nonparoxysmal atrial fibrillation patients: Results from the randomized oasis trial. J. Am. Coll. Cardiol. 68, 274–282. 10.1016/j.jacc.2016.04.015 27163758

[B44] Narayan S. M. Baykaner T. Clopton P. Schricker A. Lalani G. G. Krummen D. E.. (2014). Ablation of rotor and focal sources reduces late recurrence of atrial fibrillation compared with trigger ablation alone: extended follow-up of the confirm trial (conventional ablation for atrial fibrillation with or without focal impulse and rotor modulation). J. Am. Coll. Cardiol. 63, 1761–1768. 10.1016/j.jacc.2014.02.543 24632280 PMC4008643

[B45] Narayan S. M. Jalife J. (2014a). Crosstalk proposal: rotors have been demonstrated to drive human atrial fibrillation. J. Physiol. 592, 3163–3166. 10.1113/jphysiol.2014.271031 25085968 PMC4146362

[B46] Narayan S. M. Jalife J. (2014b). Rebuttal from Sanjiv M. Narayan and José Jalife. J. Physiol. 592:3171. 10.1113/jphysiol.2014.275396 25085970 PMC4146364

[B47] Narayan S. M. Krummen D. E. Shivkumar K. Clopton P. Rappel W.-J. Miller J. M. (2012). Treatment of atrial fibrillation by the ablation of localized sources: confirm (conventional ablation for atrial fibrillation with or without focal impulse and rotor modulation) trial. J. Am. Coll. Cardiol. 60, 628–636. 10.1016/j.jacc.2012.05.022 22818076 PMC3416917

[B48] Newman M. (2010). Networks: An Introduction. New York, NY: Oxford University Press Inc.

[B49] Oesterlein T. G. Loewe A. Lenis G. Luik A. Schmitt C. Doessel O. (2018). Automatic identification of reentry mechanisms and critical sites during atrial tachycardia by analyzing areas of activity. IEEE Trans. Biomed. Eng. 65, 2334–2344. 10.1109/TBME.2018.2794321 29993521

[B50] Patel A. M. d'Avila A. Neuzil P. Kim S. J. Mela T. Singh J. P.. (2008). Atrial tachycardia after ablation of persistent atrial fibrillation: identification of the critical isthmus with a combination of multielectrode activation mapping and targeted entrainment mapping. Circulation 1, 14–22. 10.1161/CIRCEP.107.748160 19808389

[B51] R Core Team (2017). R: A Language and Environment for Statistical Computing. Vienna: R Foundation for Statistical Computing.

[B52] Richter U. Faes L. Ravelli F. Sornmo L. (2012). Propagation pattern analysis during atrial fibrillation based on sparse modeling. IEEE Trans. Biomed. Eng. 59, 1319–1328. 10.1109/TBME.2012.2187054 22328169

[B53] Rostock T. Drewitz I. Steven D. Hoffmann B. A. Salukhe T. V. Bock K.. (2010). Characterization, mapping, and catheter ablation of recurrent atrial tachycardias after stepwise ablation of long-lasting persistent atrial fibrillation. Circulation 3, 160–169. 10.1161/CIRCEP.109.899021 20133933

[B54] Sizemore A. E. Giusti C. Kahn A. Vettel J. M. Betzel R. F. Bassett D. S. (2018). Cliques and cavities in the human connectome. J. Comput. Neurosci. 44, 115–145. 10.1007/s10827-017-0672-6 29143250 PMC5769855

[B55] Spach M. S. Dolber P. C. (1986). Relating extracellular potentials and their derivatives to anisotropic propagation at a microscopic level in human cardiac muscle. Evidence for electrical uncoupling of side-to-side fiber connections with increasing age. Circul. Res. 58, 356–371. 10.1161/01.RES.58.3.356 3719925

[B56] Spach M. S. Miller W. T. Miller-Jones E. Warren R. B. Barr R. C. (1979). Extracellular potentials related to intracellular action potentials during impulse conduction in anisotropic canine cardiac muscle. Circul. Res. 45, 188–204. 10.1161/01.RES.45.2.188 445703

[B57] Stam C. J. (2014). Modern network science of neurological disorders. Nat. Rev. Neurosci. 15, 683–695. 10.1038/nrn3801 25186238

[B58] Taccardi B. Punske B. B. Sachse F. Tricoche X. Colli-Franzone P. Pavarino L. F.. (2005). Intramural activation and repolarization sequences in canine ventricles. experimental and simulation studies. J. Electrocardiol. 38, 131–137. 10.1016/j.jelectrocard.2005.06.099 16226088

[B59] Tao S. Way S. F. Garland J. Chrispin J. Ciuffo L. A. Balouch M. A.. (2017). Ablation as targeted perturbation to rewire communication network of persistent atrial fibrillation. PLoS ONE 12:e0179459. 10.1371/journal.pone.0179459 28678805 PMC5497967

[B60] ten Tusscher K. H. Panfilov A. V. (2006). Alternans and spiral breakup in a human ventricular tissue model. Am. J. Physiol. Heart Circ. Physiol. 291, H1088–H1100. 10.1152/ajpheart.00109.2006 16565318

[B61] Thomas Lumley V. J. C. P. Ripley B. (2015). gee: Generalized Estimation Equation Solver. R package version 4.13-19.

[B62] Tomii N. Yamazaki M. Arafune T. Honjo H. Shibata N. Sakuma I. (2016). Detection algorithm of phase singularity using phase variance analysis for epicardial optical mapping data. IEEE Trans. Biomed. Eng. 63, 1795–1803. 10.1109/TBME.2015.2502726 26599526

[B63] Tusscher K. H. Hren R. Panfilov A. V. (2007). Organization of ventricular fibrillation in the human heart. Circul. Res. 100, e87–101. 10.1161/CIRCRESAHA.107.150730 17540975

[B64] Tusscher K. H. Panfilov A. V. (2003). Reentry in heterogeneous cardiac tissue described by the luo-rudy ventricular action potential model. Am. J. Physiol. Heart Circul. Physiol. 284, H542–H548. 10.1152/ajpheart.00608.2002 12388228

[B65] Umapathy K. Nair K. Masse S. Krishnan S. Rogers J. Nash M. P.. (2010). Phase mapping of cardiac fibrillation. Circulation 3, 105–114. 10.1161/CIRCEP.110.853804 20160178

[B66] Van Nieuwenhuyse E. Seemann G. Panfilov A. V. Vandersickel N. (2017). Effects of early afterdepolarizations on excitation patterns in an accurate model of the human ventricles. PLoS ONE 12:e0188867. 10.1371/journal.pone.0188867 29216239 PMC5720514

[B67] Vandersickel N. Bossu A. De Neve J. Dunnink A. Meijborg V. M. F. van der Heyden M. A. G.. (2017). Short-lasting episodes of torsade de pointes in the chronic atrioventricular block dog model have a focal mechanism, while longer-lasting episodes are maintained by re-entry. JACC 3, 1565–1576. 10.1016/j.jacep.2017.06.016 29759839

[B68] Vandersickel N. Kazbanov I. V. Nuitermans A. Weise L. D. Pandit R. Panfilov A. V. (2014). A study of early afterdepolarizations in a model for human ventricular tissue. PLoS ONE 9:e84595. 10.1371/journal.pone.0084595 24427289 PMC3888406

[B69] Vandersickel N. Van Nieuwenhuyse E. Van Cleemput N. Goedgebeur J. El Haddad M. De Neve J. (2019). Directed networks as a novel way to describe and analyze cardiac excitation: Directed graph mapping. bioRxiv 596288. 10.1101/596288 PMC6746922 31551814

[B70] Vijayakumar R. Vasireddi S. K. Cuculich P. S. Faddis M. N. Rudy Y. (2016). Methodology considerations in phase mapping of human cardiac arrhythmias. Circulation 9:e004409. 10.1161/CIRCEP.116.004409 27906655 PMC5137810

[B71] Weerasooriya R. Khairy P. Litalien J. Macle L. Hocini M. Sacher F.. (2011). Catheter ablation for atrial fibrillation: are results maintained at 5 years of follow-up? J. Am. Coll. Cardiol. 57, 160–166. 10.1016/j.jacc.2010.05.061 21211687

[B72] Weiss J. N. Qu Z. Shivkumar K. (2016). Ablating atrial fibrillation: a translational science perspective for clinicians. Heart Rhythm 13, 1868–1877. 10.1016/j.hrthm.2016.05.026 27241354 PMC4996702

[B73] White D. R. Borgatti S. P. (1994). Betweenness centrality measures for directed graphs. Soc. Netw. 16, 335–346. 10.1016/0378-8733(94)90015-9

[B74] Zahid S. Whyte K. N. Schwarz E. L. Blake R. C. Boyle P. M. Chrispin J.. (2016). Feasibility of using patient-specific models and the “minimum cut” algorithm to predict optimal ablation targets for left atrial flutter. Heart Rhythm 13, 1687–1698. 10.1016/j.hrthm.2016.04.009 27108938 PMC5972526

[B75] Zeemering S. Peeters R. van Hunnik A. Verheule S. Schotten U. (2013). Identification of recurring wavefront propagation patterns in atrial fibrillation using basis pursuit, in Engineering in Medicine and Biology Society (EMBC), 2013 35th Annual International Conference of the IEEE (Osaka: IEEE), 2928–2931. Available online at: https://ieeexplore.ieee.org/document/6610153 10.1109/EMBC.2013.6610153 24110340

[B76] Zoni-Berisso M. Lercari F. Carazza T. Domenicucci S. (2014). Epidemiology of atrial fibrillation: European perspective. Clin. Epidemiol. 6:213. 10.2147/CLEP.S47385 24966695 PMC4064952

