# Supplementary material for: Directed Networks as a Novel Way to Describe and Analyze Cardiac Excitation: Directed Graph Mapping
Source: Front Physiol. 2019 Sep 10;10:1138. doi: 10.3389/fphys.2019.01138 (PMC6746922; doi:10.3389/fphys.2019.01138)
Supplement: Supplementary file 1 [file Data_Sheet_1.pdf]

Supplementary material

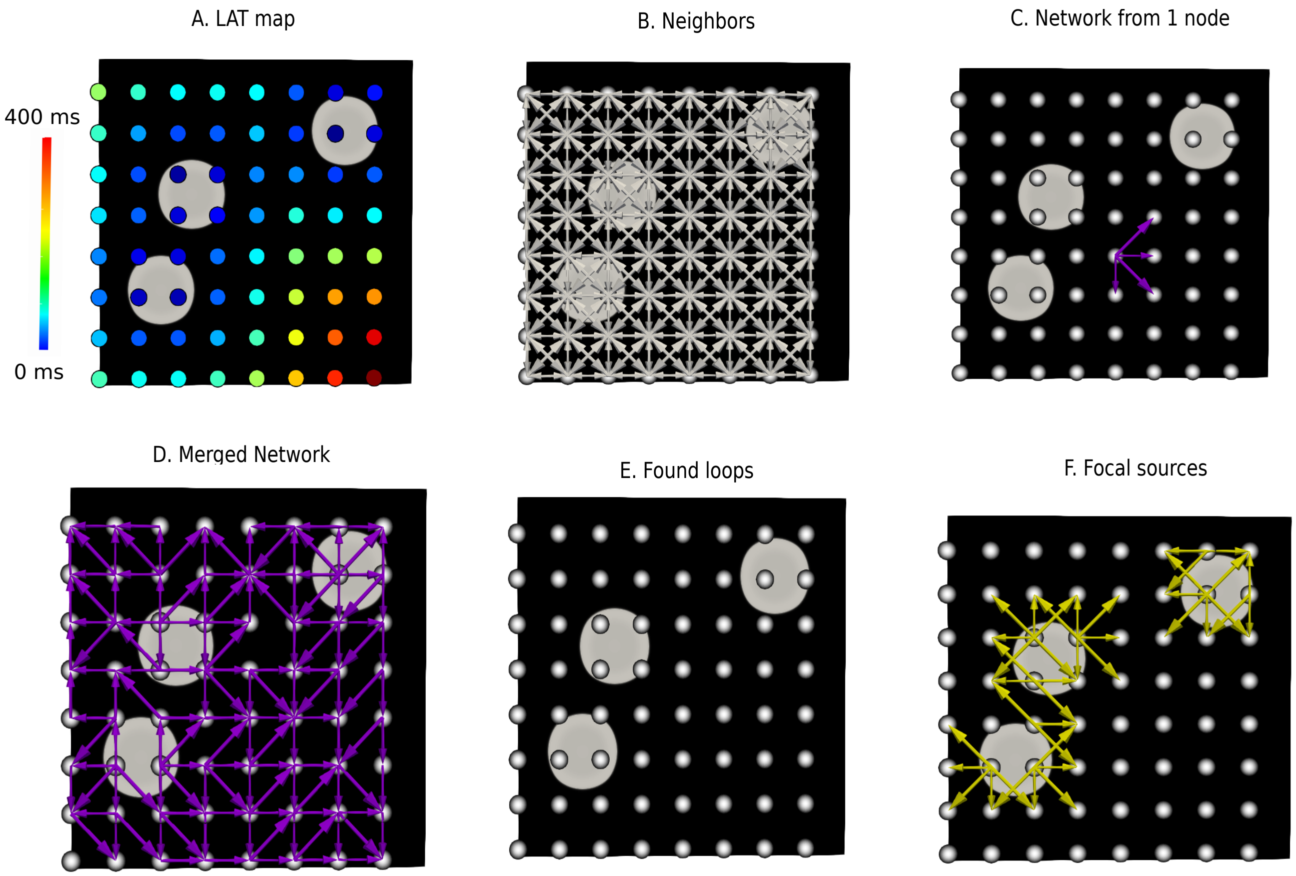

Fig. S1. DG mapping for 3 simulated focal sources with a regular measuring grid. In panel A, the points are colored according to their LAT. Panel B shows the possible neighbors (maximum 8) for each grid point. Panel C represents the corresponding network for a single node. In Panel D the complete network is drawn. No loops were detected in this network, see panel E. The focal sources are shown in panel F.

#	code	# el	CL	Mechanism LAT map CARTO (EP)	Mechanism DG
1	ATM1	865	250	Macro reentry: perimitral circuit clockwise	✓
2	ATM2	784	300	Hypothesis 1: Large macro reentrant circuit sticking close to the right veins, traveling up on the anterior wall, over the roof and down on the posterior wall. There is a SCC on the anterior RSPV side.<br>Hypothesis 2: localized reentry circuit at the anterior wall next to the RSPV through the SCC<br>ablation was done on the SCC, which agrees with both hypothesis.	✓(hypothesis 2)
3	ATM3	219	450	Hypothesis 1: Large macro reentry: roof traveling up on the anterior wall, over the roof and down on the posterior wall.<br>Hypothesis 2: localized reentry on the anterior wall.	✓(hypothesis 1)
4	ATM4	1722	225	Double loop: macro reentry: perimitral reentry counterclockwise and macro reentry around the LV over the roof.	✓
5	ATM5	1567	200	Macro reentry: perimitral reentry clockwise	✓

6	ATM6	1752	214	Hypothesis 1: double loop: macro reentry: perimitral counterclockwise and macro reentry around the LV over the roof.<br>Hypothesis 2: roof dependent macro reentry, with a pseudo (slower) reentry around the mitral valve.	Only perimitral counter-clockwise reentry found but the ablation target would be at the same location ✓
7	PC1	1753	245	Focal	✓(no loops detected)
8	PC2	889	290	Macro reentry: roof	✓
9	PC3	896	302	Macro reentry: perimitral reentry clockwise	✓
10	PC4	431	240	Perimitral circuit counterclockwise. This was ablated and the AT changed and switched through the roof with a CL of 228 ms. However, retrospective offline analysis revealed a localized reentry below the left atrial appendage (at the base of the LAA). Original ablation points agree with localized reentry.	✓(localized-reentry)
11	PC5	847	250	Macro reentry: peritricuspid reentry in the right atrium	✓
12	PC6	444	278	Macro reentry: mitral reentry counterclockwise	✓
13	PC7	354	340	Macro reentry: perimitral circuit	✓
14	PC8	938	260	Macro reentry: perimitral circuit counterclockwise and a second loop going through the roof. The two loops are merging at the left veins.	Only perimitral reentry found but the ablation target would be at the same location ✓
15	PC9	283	304	Focal	✓(no loops detected)
16	PC10	3524	240	The online LAT map was uninterpretable, however roof line ablation was performed. Offline retrospective re-evaluation showed a possible atypical focal activity from the scar on the roof. The ablation roof line went through that scar and that possible terminated the AT.	✓(no loops detected)
17	CD201	475	260	Localized localized reentry at the crosspoint at the appendage and the roof, anterior superior appendage from the left atrium.	✓
18	CD203	1092	240	Localized localized reentry at the anterior wall near the mitral annulus at 11 o' clock.	✓
19	CD204	1091	240	Hypothesis 1: macro reentry: perimitral circuit counterclockwise using a SCC in between the mitral anulus at 10 o'clock and a scar at the anterior wall.<br>Hypothesis 2: the same loop as in hypothesis 1, but a second loop is found through the same SCC turning around the scar at the anterior wall.<br>Ablation at the SCC terminated the AT (according to hp1).	✓ (hypothesis 1)
20	CD205	463	290	Hypothesis 1: macro reentry: perimitral circuit clockwise using a SCC in between the mitral annulus at 10'o clock and a scar at the anterior wall.<br>Hypothesis 2: the same loop as in hypothesis 1, but a second loop through the same SCC turning around the scar at the anterior wall counterclockwise. (the size of this second loop cannot be determined).<br>Ablation at the SCC terminated the AT (according to hypothesis 1)	✓ (hypothesis 1).
21	CD206a	612	250	Focal breakthrough at the septum inferior and right inferior PV.	✓(no loops detected)
22	CD206b	360	250	Focal AT at posterior septum superior, but 2 signals were not able to be explained by the focal activity.<br>After diagnosis from DG mapping, the map was reconsidered and localized reentry agreed better with the map.	Localized reentry at the posteral septal wall but the ablation target would be at the same location ✓
23	CD207	648	215	Macro reentry: clockwise mitral valve	✓

24	CD208	876	315	Hypothesis 1: Macro reentry circuit at the septum anterior from the right superior pulmonary vain, active or passive.<br>Hypothesis 2: Same loop as in hypothesis 1, but it might be passive because of another loop with the same pathway that turns around the left veins through the mitral isthmus, coming back over the roof. Ablation at the roof terminated the AT.	✓(hypothesis 1)
25	CD209	489	270	Macro reentry: roof ascending over the anterior wall and descending over the posterior wall.	✓
26	CD210	628	340	Localized reentry at the septum	✓
27	CD211	603	210	Hypothesis 1: Localized reentry or might be larger reentry at the anterior wall near the mitral annulus at 11 o'clock anterior from the LAA.<br>Hypothesis 2: Counterclockwise perimitral circuit: less likely because of colliding wavefront. EP treated as hypothesis 1.	✓(hypothesis 1)
28	CD212a	1147	250	Hypothesis 1: Macro reentry tachycardia roof dependent, ascending on the anterior wall, descending on posterior wall, collision on posterior mitral isthmus.<br>Hypothesis 2: it could be a smaller loop clockwise around perimitral.	✓(hypothesis 1)
29	CD212b	1089	285	Macro reentry: clockwise mitral valve	✓
30	CD213	1322	305	There is a SCC at the roof near the RV (there is a gap in a prior ablation line).<br>Hypothesis 1: through SCC a large macro reentry: around the RV.<br>Hypothesis 2: through SCC, more localized reentrant circuit using a second gap at the roof for reentry.<br>Hypothesis 3: Both hypothesis might be correct (i.e. double loop).<br>EP ablated at the SCC at the roof, according to hypothesis 1.<br>EP ablated at the SCC at the roof, according to hypothesis 1.	✓(hypothesis 1)
31	CD214	439	305	Macro reentry: perimitral reentry clockwise pushed away from the mitral annulus (Y shape).	✓

Table S1. Summary of the 31 clinical cases of regular AT. For cases 1-16, we used non-interpolated data given by the CARTO system. For cases 17-32, the interpolated data from CARTO was used. The columns represent the case number (#), the database code, the number of measured points (# el), the cycle length (CL), the mechanism found by the EP and the mechanism found by DG mapping. We put a ✓if the mechanism fully agrees, and a ✓ if the ablation point agrees, but a different mechanism is seen. SCC = slow conductive channel, PV = pulmonary vain, RSPV = right superior pulmonary vain, RV = right vain, LAA = Left Anterior Appendage.

	64				256			
	0.5		1		0.5		1	
σ	PM	DG	PM	DG	PM	DG	PM	DG
0	100	100	100	100	100	100	100	100
5	73	45	92	96	68	98	74	100
10	46	40	75	91	49	95	62	100
15	26	35	51	83	19	83	30	95
20	9	27	21	68	0	62	1	81
25	2	18	6	50	0	40	0	60
30	1	12	2	34	0	24	0	41

Table S2. Percentage of correct cores as a function of increasing noise (i.e. increasing standard deviation σ of the Gaussian noise) for varying radius to the true core (0.5 or 1 cm) and a varying number of electrodes (64 or 256). PM stands for phase mapping and DG for DG mapping. DG mapping outperforms phase mapping except for a radius of 0.5 cm with 64 electrodes.

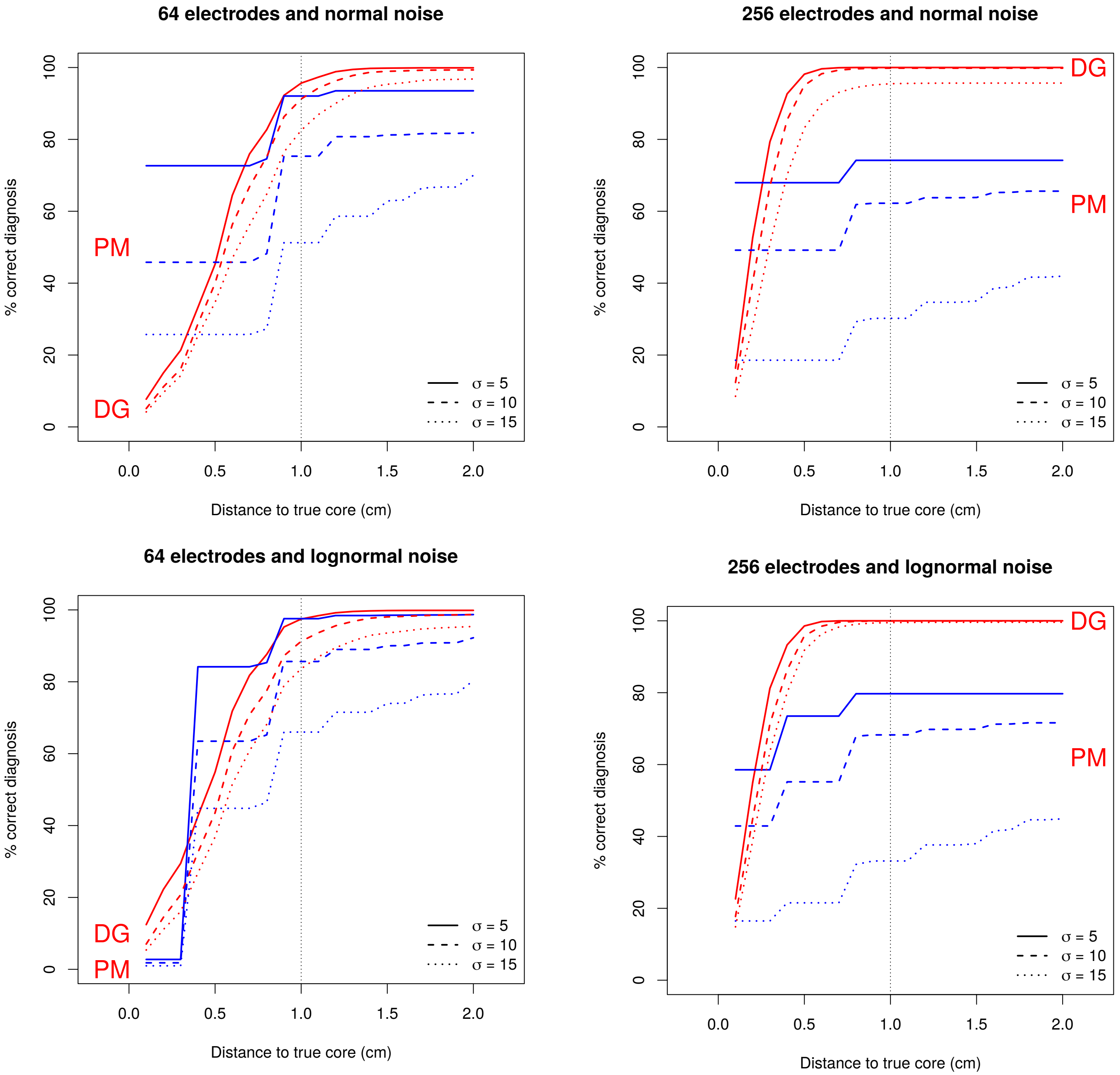

Fig. S2. Percentage of correct diagnoses as a function of distance to the true core for different levels of the standard deviation of the noise σ. The red lines denote DG mapping the blue lines phase mapping (PM). The top panels show the performance for normal noise, while for the bottom panels the noise follows a lognormal distribution. DG mapping has a continuous behavior, while phase mapping shows a stepwise behavior. This is because phase mapping has a resolution determined by the size of the smallest square in the tissue (16x16 mm for the 64 electrode grid, 8x8 mm for the 265 electrode). The minimal steps in performance are seen each 8 mm for 64 electrodes and 4 mm for 265 electrodes, which agrees with the difference of the center of one square and the average of the centers of two contiguous squares, as shown in Figure S3. Therefore, the minimal step in accuracy for phase mapping is determined by the grid size. Phase mapping and DG mapping can only be compared above this minimal accuracy. The graphs display a superior performance of DG mapping over phase mapping for both normal and lognormal noise, for all choices of σ and for distances to the true core exceeding 1 cm. Overall, the performance of DG mapping is similar for normal and lognormal noise and improves with increasing number of electrodes.

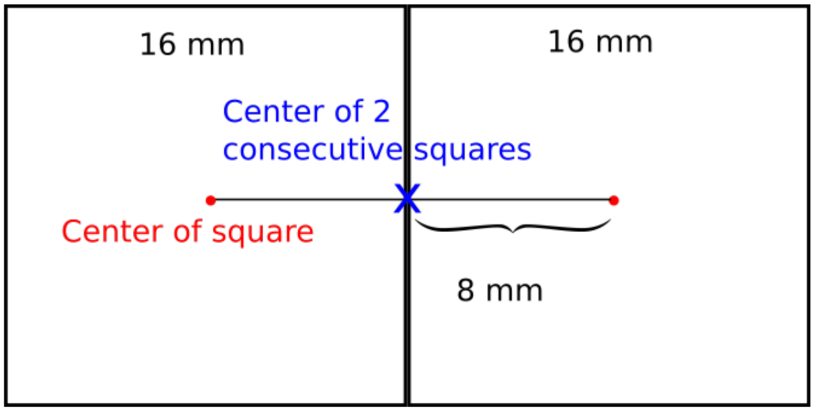

Fig. S3. Minimal distance between the center of a square and two consecutive squares for 64 electrodes.
